# Supplementary material for: Analyses of lncRNAs, circRNAs, and the Interactions between ncRNAs and mRNAs in Goat Submandibular Glands Reveal Their Potential Function in Immune Regulation
Source: Genes (Basel). 2023 Jan 10;14(1):187. doi: 10.3390/genes14010187 (PMC9859278; doi:10.3390/genes14010187)
Supplement: Supplementary file 1 [file genes-14-00187-s001.zip › Table S1 LncRNA transcript statistics from different sample groups.docx]

Table S1 LncRNA transcript statistics from different sample groups

| Group name | All isoform num | Known isoform num | New isoform num |
| --- | --- | --- | --- |
| A | 3,192 | 1,627 （60.80%） | 1,565 |
| B | 2,936 | 1,492 （55.75%） | 1,444 |
| C | 2,730 | 1,381 （51.61%） | 1,349 |
