# Supplementary material for: Analyses of lncRNAs, circRNAs, and the Interactions between ncRNAs and mRNAs in Goat Submandibular Glands Reveal Their Potential Function in Immune Regulation
Source: Genes (Basel). 2023 Jan 10;14(1):187. doi: 10.3390/genes14010187 (PMC9859278; doi:10.3390/genes14010187)
Supplement: Supplementary file 1 [file genes-14-00187-s001.zip › Table S2 LncRNA transcript statistics from each sample.docx]

Table S2 LncRNA transcript statistics from each sample

| Sample Name | Known Isoform Num | New Isoform Num | All Isoform Num |
| --- | --- | --- | --- |
| A1-L | 1,228 （45.89%） | 1,348 | 2,576 |
| A2-L | 1,002 （37.44%） | 1,064 | 2,066 |
| A3-L | 1,291 （48.24%） | 1,389 | 2,680 |
| B3-L | 1,128 （42.15%） | 1,221 | 2,349 |
| B4-L | 1,080 （40.36%） | 1,132 | 2,212 |
| B5-L | 1,087 （40.62%） | 1,171 | 2,258 |
| C2-L | 1,072 （40.06%） | 1,140 | 2,212 |
| C3-L | 896 （33.48%） | 1,023 | 1,919 |
| C5-L | 861 （32.17%） | 959 | 1,820 |
