# Supplementary material for: Analyses of lncRNAs, circRNAs, and the Interactions between ncRNAs and mRNAs in Goat Submandibular Glands Reveal Their Potential Function in Immune Regulation
Source: Genes (Basel). 2023 Jan 10;14(1):187. doi: 10.3390/genes14010187 (PMC9859278; doi:10.3390/genes14010187)
Supplement: Supplementary file 1 [file genes-14-00187-s001.zip › Table S3 Length statistics of lncRNAs.docx]

Table S3 Length statistics of lncRNAs

| Type | Length/nt | Number | Ratio | Sum |
| --- | --- | --- | --- | --- |
| all lncRNA | 301-400 | 274 | 6.22% | 4,404 |
|  | 200-1,200 | 2,245 | 50.98% |  |
| novel lncRNA | 501-600 | 97 | 5.61% | 1,728 |
|  | 301-1,300 | 760 | 49.13% |  |
|  | 1,601-1,900 | 89 |  |  |
| DElncRNA | 1,101-1,200 | 20 | 5.42% | 369 |
|  | 1,101-1,900 | 97 | 51.36% |  |
|  | 400-900 | 60 |  |  |
