# Supplementary material for: Analyses of lncRNAs, circRNAs, and the Interactions between ncRNAs and mRNAs in Goat Submandibular Glands Reveal Their Potential Function in Immune Regulation
Source: Genes (Basel). 2023 Jan 10;14(1):187. doi: 10.3390/genes14010187 (PMC9859278; doi:10.3390/genes14010187)
Supplement: Supplementary file 1 [file genes-14-00187-s001.zip › Table S4 Statistics on the number of mRNA, lncRNA, and miRNA with potential regulatory relationships with circRNA.doc]

Table S4 Statistics on the number of mRNA, lncRNA, and miRNA with potential regulatory relationships with circRNA

| Type | ncRNA num | mRNA num | Pairs num | miRNA num |
| --- | --- | --- | --- | --- |
| lncRNA | 318 | 2,208 | 48,162 | 238 |
| circRNA | 698 | 2,273 | 63,357 | 253 |
| total | 1,016 | 2,292 | 111,519 | 260 |
