# Supplementary material for: Analyses of lncRNAs, circRNAs, and the Interactions between ncRNAs and mRNAs in Goat Submandibular Glands Reveal Their Potential Function in Immune Regulation
Source: Genes (Basel). 2023 Jan 10;14(1):187. doi: 10.3390/genes14010187 (PMC9859278; doi:10.3390/genes14010187)
Supplement: Supplementary file 1 [file genes-14-00187-s001.zip › Table S5 Information statistics for circRNAs that have an adsorption relationship and a high correlation with miR-141-x, miR-29-y and chi-miR-29b-3p.doc]

Table S5 Information statistics for circRNAs that have an adsorption relationship and a high correlation with miR-141-x, miR-29-y and chi-miR-29b-3p

| miRNA | circRNA | Pearson correlation | Source-gene of circRNA | Chromosome | type |
| --- | --- | --- | --- | --- | --- |
| miR-141-x | novel_circ_001701 | -.991** | ncbi_102183190 | NC_030809.1 | annot_exons |
|  | novel_circ_016795 | -.930** | ncbi_102185993 | NW_017189516.1 | annot_exons |
|  | novel_circ_017078 | -.919** | ncbi_106503784 | NW_017189517.1 | annot_exons |
|  | novel_circ_002843 | -.913** | / | NC_030811.1 | intergenic |
|  | novel_circ_016987 | -.903** | ncbi_102189748 | NW_017189516.1 | exon_intron |
|  | novel_circ_005714 | -.902** | ncbi_102186582 | NC_030814.1 | annot_exons |
| miR-29-y | novel_circ_010888 | -.988** | ncbi_102168652 | NC_030822.1 | annot_exons |
|  | novel_circ_004648 | -.914** | ncbi_102169466 | NC_030813.1 | annot_exons |
|  | novel_circ_003202 | -.904** | ncbi_102175459 | NC_030811.1 | exon_intron |
|  | novel_circ_017147 | -.903** | / | NW_017189541.1 | intergenic |
| chi-miR-29b-3p | novel_circ_002837 | -.927** | / | NC_030811.1 | intergenic |
|  | novel_circ_010888 | -.923** | ncbi_102168652 | NC_030822.1 | annot_exons |
|  | novel_circ_002834 | -.917** | / | NC_030811.1 | intergenic |
|  | novel_circ_012944 | -.911** | ncbi_102188081 | NC_030826.1 | annot_exons |
|  | novel_circ_017147 | -.911** | / | NW_017189541.1 | intergenic |

** represents a significant correlation at level 0.01 (double-tailed)，* epresents a significant correlation at level 0.05 (double-tailed)
