# Supplementary material for: Analyses of lncRNAs, circRNAs, and the Interactions between ncRNAs and mRNAs in Goat Submandibular Glands Reveal Their Potential Function in Immune Regulation
Source: Genes (Basel). 2023 Jan 10;14(1):187. doi: 10.3390/genes14010187 (PMC9859278; doi:10.3390/genes14010187)
Supplement: Supplementary file 1 [file genes-14-00187-s001.zip › Table S6 Information statistics for lncRNAs that have an adsorption relationship and a high correlation with miR-141-x, miR-29-y and chi-miR-29b-3p.doc]

Table S6 Information statistics for lncRNAs that have an adsorption relationship and a high correlation with miR-141-x, miR-29-y and chi-miR-29b-3p

| miRNA | lncRNA | Pearson correlation |
| --- | --- | --- |
| miR-141-x | TCONS_00029100 | -.956** |
|  | TCONS_00013433 | -.935** |
|  | TCONS_00005821 | -.934** |
|  | TCONS_00016710 | -.921** |
|  | TCONS_00059228 | -.914** |
|  | TCONS_00021591 | -.909** |
| miR-29-y | TCONS_00028293 | -.940** |
|  | TCONS_00039798 | -.940** |
|  | TCONS_00021591 | -.932** |
|  | TCONS_00013433 | -.929** |
|  | TCONS_00072313 | -.926** |
|  | TCONS_00003575 | -.925** |
|  | TCONS_00040873 | -.922** |
|  | XR_001918722.1 | -.918** |
|  | TCONS_00010787 | -.914** |
|  | XR_001919759.1 | -.910** |
| chi-miR-29b-3p | TCONS_00028293 | -.935** |
|  | TCONS_00013433 | -.929** |
|  | TCONS_00039798 | -.929** |
|  | TCONS_00059228 | -.922** |
|  | TCONS_00072313 | -.918** |
|  | TCONS_00003575 | -.906** |
|  | XR_001919759.1 | -.903** |
|  | XR_001918722.1 | -.902** |

** represents a significant correlation at level 0.01 (double-tailed)，* represents a significant correlation at level 0.05 (double-tailed)
